# Binge eating, sociodemographic and lifestyle factors in participants of the ELSA-Brazil

**DOI:** 10.1186/s40337-016-0095-1

**Published:** 2016-10-27

**Authors:** Thamyres Souza da Silva, Maria Del Carmen Bisi Molina, Maria Angélica Antunes Nunes, Carolina Perim de Faria, Nagela Valadão Cade

**Affiliations:** 1 Federal University of Espírito Santo (Universidade Federal do Espírito Santo), Vitória, Brazil; 2 Federal University of Rio Grande do Sul (Universidade Federal do Rio Grande do Sul), Porto Alegre, Brazil

**Keywords:** Binge Eating Disorder, Eating Behaviour, Lifestyle, Obesity

## Abstract

**Background:**

This study investigates the relationship between recurrent binge eating episodes and nutritional and food profiles and lifestyle in the Brazilian Longitudinal Study of Adult Health (Estudo Longitudinal da Saúde do Adulto – ELSA-Brazil) cohort.

**Results:**

Recurrent binge eating episodes were associated with obesity (OR 5.188; confidence interval [CI] 4.051–6.645), overweight (OR 2.534; CI 1.980–3.243), female sex (OR 1.918; CI 1.573–2.338), age between 34 and 54 years old (OR 1.349; CI 1.115–1.631), alcohol ingestion ≥ 5 in two hours (OR 1.397; CI 1.068–1.827), and insufficient physical activity (OR 1.290; CI 1.078–1.544).

**Conclusion:**

Being overweight has an important association with recurrent binge eating episodes, as does demographic and lifestyle characteristics, including excessive alcohol consumption.

## Background

Binge eating is a behaviour characterised by exaggerated food ingestion over a short period of time, followed by a sense of loss of control over the amounts eaten [1–4]. Binge eating behaviour occurs as a central symptom of eating disorders such as binge eating disorder (BED) bulimia nervosa and anorexia nervosa or as sporadic, a partial behavior when they do not meet all the diagnostic criteria for the disorder, but it can bring discomfort and seeking treatment because of the recurrent binge eating episodes [2, 3].

Studies performed among the Brazilian population have reported the prevalence of recurrent binge eating episodes to range from 12.8 % in the general population over 18 years [5], 24.6 % in teenagers, with girls presenting a higher prevalence than boys (31 %) [6] and up to 39.3 % in overweight adults [7].A study performed by Hood et al. found a prevalence of recurrent binge eating episodes of 33 % among obese adults, which was similar to the Brazilian studies [8]. Although different methods of assessment have been used, generally similar prevalences of recurrent binge eating episodes are reported worldwide in obese and general population groups [9–11].

Individuals with recurrent binge eating episodes or BED present with higher levels of caloric consumption [12] and cravings (abnormal food desires) [13]. Other factors not diet-related that are associated with BED include higher alcohol consumption [6, 14], lower physical activity [6, 15], anxiety and depression [16] and health conditions such as diabetes and hypertension [17].

The goal of the present study was to investigate the frequency of recurrent binge eating episodes and their relationship with nutritional profiles, eating profiles and selected lifestyle factors in a cohort of 15,105 public servants aged between 35 and 74 years old from the Brazilian Longitudinal Study of Adult Health (Estudo Longitudinal da Saúde do Adulto – ELSA-Brazil). The present study is important because few studies have investigated the prevalence of recurrent binge eating episodes and related risk factors in adult, aging populations and in the non-obese [5, 8, 9, 11, 16, 18, 19].

## Method

### Design and participants

This cross sectional observational study used baseline data from the Brazilian Longitudinal Study of Adult Health (ELSA-Brazil) [20]. The sample consisted of 15,105 public servants from six higher education institutions, active or retired, aged between 35 and 74 years old, who participated in the first stage of data collection of ELSA-Brazil, which ended in 2010.

The outcome variable was the presence of recurrent binge eating episodes, and the exposure variables included sociodemographic (sex, age, self-reported race/ethnicity, level of education and income per capita), nutritional (total calories and calories per food group), weight (body mass index [BMI]), and other lifestyle (smoking, alcohol and physical activity) factors. In Brazil, due to a wide variety of mixed national and ethnic groups in the population, race/ethnicity is categorised using self-reported skin color of black, brown, white, yellow (Asian) or red (indigenous).

### Assessments

Recurrent binge eating episodes evaluation was based on the DSM IV [3] definition as in the following question: “*Some people, in some occasions, eat large amounts of food at once, over a short period of time (up to 2 hours). They feel that they have lost control, that is, they cannot avoid starting to eat, and after they start, they cannot stop. Over the past six months, how frequently did you eat in this way?”* recurrent binge eating episodes were considered present when a participant reported that this type of overeating occurred twice or more a week over the previous six months. Education was categorized according to the schooling level: first degree or fundamental (primary) up to nine years of study, high school up to 12 years of study and college (university) over 12 years of study, and monthly income and income per capita, calculated based on the value of minimum wage at the time of the study.

Food consumption data were collected using the Food Frequency Questionnaire (FFQ. This semi-quantitative tool includes 114 items and focuses on the usual ingestion profile over the last twelve months [21].

Energy consumption was evaluated using Nutrition Data System for Research (NDRS) software. Overestimation due to self-referring was corrected by adjusting the value to the 99 percentile, and when seasonal food consumption was observed, the total value of daily consumption was multiplied by 0.25.

Alcohol consumption (in grams of ethanol) data were obtained from the FFQ; it was also assessed by a specific questionnaire that focused on drinking habits and the frequency of consumption of five or more drinks of any kind in periods of two hours regardless of the frequency, suggests episodes of compulsive drinking [22].

Smoking was evaluated using a semi-structured questionnaire with questions about smoking habits at the time of the interview, in the past, or whether the participant had never smoked [19]. Physical activity was evaluated using the International Physical Activity Questionnaire (IPAQ) [23], with previous validity having been demonstrated [24].

Activity levels took account of the sum of the activities related to leisure and commuting, as currently recommended [25].

Height and weight were measured using a stadiometer (SECA-SE-216) with a 0.1 cm scale and an electronic scale (Toledo 2096 PP, measures up to 200 kg). BMI was calculated from the height and weight values, with these measurements taken with participants dressed in standard clothes and without shoes or glasses [26]. The BMI cut-off points proposed by the World Health Organization (2000) were used as a reference [27].

All data were collected by personnel trained and certified for the use of standardised ELSA protocols, face-to-face interview questionnaires, and benchmarking.

### Statistical analysis

Categorical variables were compared using the Chi-square test, Fisher's exact test, or the maximum likelihood ratio. Differences between groups of non-parametric continuous data were tested using the Mann-Whitney U test. Thereafter logistic regression was performed to identify significant univariate factors uniquely associated with recurrent binge eating episodes. Results of the bivariate analysis were used to determine which variables were inserted on the final model.

Odds ratios (OR) with a 95 % confidence interval (CI) were calculated. All analyses were performed using SPSS 15.0 software, with significance set at *p* < 0.05.

### Ethics

Because this study was a multicentre study, the ELSA-Brazil project was approved by the Research Ethics National Committee (Comitê Nacional de Ética em Pesquisa) and by the committees of each institution involved in December 2008 (Study registration number = 140/08).

## Results

Among the 15,074 participants (99.9 % of the ELSA-Brazil) who answered the question regarding compulsion, 980 (6.5 %; 95 % CI 6.1–6.9 %) participants reported binge eating episodes twice or more than twice a week, and comprised the sample of participants with recurrent binge eating episodes in this study. Of those with recurrent binge eating episodes 66.6 % were women, 68.2 % were in the younger age category, 18.7 % were of black and 30.4 % of brown race/colour and 52.9 % did not have university education. Participants with recurrent binge eating episodes were much more likely to be obese (45.9 %), to be less active (64.6 %) and to have patterns of high alcohol ingestion over short periods of time, once or twice a week (8.9 %) (Table 1).

**Table 1:** Sociodemographic, nutritional, and life style characterisation, according to the presence of recurrent binge eating episodes, in the ELSA-Brazil (*N* = 15,105)

Variables	Recurrent binge eating episodes				*p*-value
	Yes		No		
	*n*	%	*n*	%	
Sex					
Male	327	33.4	6548	45.6	
Female	653	66.6	7546	54.4	0.000
Total	980		14,094		
Age					
34 to 54 years old	668	68.2	8597	61.0	
55 to 75 years old	312	31.8	5497	39.0	0.000
Total	980		14,094		
Colour or race					
Black	183	18.7	2211	15.7	
Brown	298	30.4	3898	27.7	
White	447	45.6	7338	52.1	0.000
Asian	21	2.1	352	2.5	
Indigenous	19	1.9	138	1.0	
Total	968		13,937		
Education level					
Incomplete primary school	70	7.1	817	5.8	
Complete primary school/incomplete secondary school	72	7.3	952	6.8	0.003
Complete secondary school/Incomplete university	377	38.5	4846	34.4	
University or Graduate	461	47.0	7479	53.1	
Total	980		14,094		
BMI					
Thin; <18.5	1	0.1	140	1.0	
Eutrophic: 18.5–24.99	144	14.7	5264	37.3	
Overweight: 25–29.99	385	39.3	5681	40.3	0.000
Obese: ≥30	450	45.9	3003	21.3	
Total	980		14,088		
Physical activity					
Insufficiently active	633	64.6	7500	53.2	
Sufficiently active	347	53.4	6594	46.8	0.000
Total	980		14,094		
Smoking					
Never smoked	528	53.9	8055	57.2	
Ex-smoker	322	32.9	4197	29.8	0.098
Smoker	130	13.3	1841	13.1	
Total	980		14,093		
Alcohol intake ≥ 5 standard units of alcohol in 2 h					
Twice a day or more	6		33	0.2	
Practically every day	3		111	0.8	
Once or twice a week	87		1072	7.6	
Twice or three times a month	43		637	4.5	0.002
Only on special occasions	236		3872	27.5	
Never	222		4103	29.1	
Total	980		14.094		

Analysis found calorie consumption was associated with weight gain (BMI). Calorie consumption was higher for participants presenting with recurrent binge eating episodes, independent of BMI (Table 2). Participants presenting with recurrent binge eating episodes also exhibited higher consumption levels in all food groups, except for group 3, vegetables and legumes, and group 8, alcoholic beverages (Table 3).

**Table 2:** Distribution of ingested calories per BMI, depending on the presence or absence of recurrent binge eating episodes, in the ELSA-Brazil (*n* = 15,105)

BMI	Binge eating	*n*	Lowest Value	Highest Value	Median	Mean	Standard deviation
Underweight; <18.5^a^	Yes	1	n.a.	n.a.	2139.05^b^	n.a.	n.a.
	No	140	1093.82	9550.04	2814.09	3182.89	1426.44
Eutrophic: 18.5–24.99	Yes	144	1080.55	15395.60	2849.63	3234.28	1644.59
	No	5259	489.71	15344.07	2648.90	2906.87	1207.48
Overweight:25–29.99	Yes	385	932.76	11673.80	2918.77	3195.73	1374.26
	No	5674	345.02	14014.68	2723.82	2959.58	1218.13
Obesity:≥30	Yes	450	961.50	11536.98	3200.46	3438.64	1380.27
	No	3002	294.02	11146.48	2744.69	2988.47	1231.65

^a^For the thin group, it was not possible to compare it with the different groups. ^b^Single value

**Table 3:** Descriptive analysis of food consumption per calories, according to the presence of recurrent binge eating episodes, in the ELSA-Brazil (*n* = 15,105)

Variables	BED	Median	Mean	Standard deviation	*p*-value*
G1: Bread, cereal and tubers	Yes	450.52	550.49	395.98	0.000
	No	370.80	449.99	323.59	
G2: Fruit	Yes	271.25	348.67	294.60	0.014
	No	255.33	317.16	251.26	
G3: Vegetables and legumes	Yes	0.00	32.12	74.62	0.001
	No	9.18	35.40	75.46	
G4: Eggs, meat, milk and derivatives	Yes	181.84	237.92	202.92	0.000
	No	162.13	205.06	169.07	
G5: Pasta and other prepared foods	Yes	165.32	198.17	147.71	0.000
	No	143.78	172.72	125.57	
G6: Sweets	Yes	88.09	135.69	143.32	0.000
	No	72.80	108.93	117.38	
G7: Non-alcoholic beverages	Yes	140.36	203.74	214.30	0.937
	No	140.27	195.52	194.25	
G8: Alcoholic beverages	Yes	8.93	56.04	119.77	0.000
	No	17.18	62.70	114.14	
Total calories	Yes	3025.08	3311.86	1422.88	0.000
	No	2704.82	2946.68	1221.28	

* Mann-Whitney test

Logistic regression analysis indicated that obese individuals presented an almost 5.2 times higher risk of binge eating episodes; overweight individuals presented a 2.5 times higher risk (Table 4). Higher recurrent binge eating episodes probabilities were present for women (1.9 times higher), individuals between 34 and 54 years old (1.4 times higher), those with alcohol ingestion higher or equal to five standard units of alcohol in two hours (1.4 times higher), and those who were insufficiently physically active (1.3 times higher).

**Table 4:** Odds ratio adjusted by the multivariate logistic regression model of lifestyle and demographic exposure variables with outcome variable recurrent binge eating episodes (*n* = 15,105)

Exposure variables	Multivariate analysis		
	*p*-value	OR	CI 95 %
Gender			
Male		1.000	–
Female	0.000	1.918	1.573–2.338
Age			
34 to 54 years old	0.002	1.349	1.115–1.631
55 to 75 years old		1.000	–
BMI			
Eutrophic		1.000	–
Thin	0.387	0.416	0.057–3.034
Overweight	0.000	2.534	1.980–3.243
Obese	0.000	5.188	4.051–6.645
Physical activity			
Insufficiently active	0.005	1.290	1.078–1.544
Sufficiently active		1.000	–
Alcohol intake ≥ 5 standard units of alcohol in 2 h			
Less frequent		1.000	–
More frequent	0.015	1.397	1.068–1.827
Calorie consumption			
Calories Group 1	0.051	1.00036	0.999–1.001
Calories Group 2	0.682	1.00009	0.99967–1.00050
Calories Group 3	0.528	0.99961	0.99841–1.00082
Calories Group 4	0.868	1.00005	0.99951–1.00058
Calories Group 5	0.281	1.00041	0.99966–1.00116
Calories Group 6	0.397	1.00031	0.99959–1.00104
Calories Group 8	0.555	0.99976	0.99895–1.00056
Total calories	0.067	1.00012	0.99999–1.00026

## Discussion

The prevalence of recurrent binge eating episodes in this study was 6.5 % and was lower than that of an earlier national study that found a prevalence of 12.8 % among adults from five Brazilian regional capitals. This difference in prevalence may be due the characteristics of the samples because the present study used a greater demographic cross-section of adults.

The results of this study indicated that being female, younger, overweight (overweight and obesity) and insufficiently physically active and using alcohol with a compulsive pattern contributed more to the occurrence of binge eating episodes. These findings are in accordance with previous studies [6, 28–33], despite differing demographic features (i.e., public servants, adults and elderly individuals, mostly with completed secondary school educations and specialised occupations).

Although few epidemiological studies describe binge eating in population representative samples, women present a higher probability of experiencing recurrent binge-eating episodes than men [28–30, 33], and is more frequent in teenagers and young adults [12, 29, 34–36]. Women and young adults may be more predisposed to recurrent binge-eating episodes and BED due to internalisation of the thin ideal [37] through which women experience overvaluation of standards of body aesthetics and weight which then can lead to eating behaviours and habits that are harmful to their health [38]. The findings are also consistent with others that have reported increases in energy intake due to high consumption of fat and sugar rich foods resulting in overweight, or decreases in daily caloric ingestion from excessive worrying about maintaining a slim and thin body in young people. Both of these may be pathways to eating disorders [39].

In this study overweight, expressed as obesity and overweight, was more strongly associated with recurrent binge eating episodes. This finding is also in accordance with previous studies that have reported that obese people to be more vulnerable to prejudice and social discrimination, which generates psychological suffering and can lead to the use of food as a compensation for problems and frustrations [40–42]. Likewise, obese individuals may isolate themselves because they feel rejected and have difficulties in obtaining pleasure from social relations. These feelings contribute to the observation that obese individuals may consider food and overeating an important source of pleasure, but which makes their affective and social relationships harder to maintain [36]. A self-perpetuating cycle of binge eating leading to higher energy consumption and increased weight, and an imbalance between energy intake and expenditure through physical activity, and adverse consequences for interpersonal function and mood can result.

Individuals with recurrent binge eating episodes presented higher caloric consumption levels from almost all food groups except for the groups of vegetables and legumes and alcoholic beverages, but recurrent binge eating episodes was not associated with over consumption on regression analyses controlling for demographic and other lifestyle variables. Although participants with recurrent binge eating episodes ingested less alcohol than those who did not have recurrent binge eating episodes, binge-drinking over a two-hour period was higher for the recurrent binge eating episode group (five or more standard units of alcohol for men and four or more for women) [3]. Previous studies have confirmed this pattern of alcohol ingestion in people presenting binge eating episodes or BED among university women [43], teenagers [6] and obese women [13]. The fact that alcohol consumption per calorie was lower for subjects with recurrent binge eating episodes may be due to an underestimation of the consumed volume by problem-drinkers or the fact that heavy drinkers have more difficulty to evaluate alcohol consumption [44, 45]. Disordered eating may be associated with alcohol abuse due to shared factors such as lack of self-control, co-morbidities such as anxiety and depression, biological predisposition for the use of psychoactive substances [40], and dissatisfaction with body image and frustrated attempts in controlling weight, which in turn could lead to excessive food and drink consumption [46].

### Strengths and limitations

A limitation in the present study is the inherent problem of comparing its findings with a literature that uses wide-ranging methodologies and samples to evaluate binge eating and food consumption. Due to the cross-sectional design, causal relationships could not be evaluated. Finally, limitations in assessment of caloric consumption with the FFQ need to be acknowledged. Although, total caloric consumption was high for recurrent binge eating episodes and non-recurrent binge eating episodes groups, the FFQ evaluates the usual consumption over the last twelve months and may be associated with over (or under) estimation of consumption due to variable perception of the portions being shown, reliance on memory recall, and interviewer skills [47].

## Conclusion

In this study, recurrent binge eating episodes were common and was associated with being overweight and/or frankly obese, and a compulsive pattern of alcohol consumption. Further studies are needed to investigate the complex and multidimensional phenomena of binge eating and its associated health consequences including obesity.
